# Palliative radiotherapy for tumor bleeding in patients with unresectable pancreatic cancer: a single-center retrospective study

**DOI:** 10.1186/s13014-023-02367-5

**Published:** 2023-10-31

**Authors:** Taro Shibuki, Mitsuhito Sasaki, Shota Yamaguchi, Kanae Inoue, Tomonao Taira, Tomoyuki Satake, Kazuo Watanabe, Hiroshi Imaoka, Shuichi Mitsunaga, Takeshi Fujisawa, Kento Tomizawa, Hidekazu Oyoshi, Masaki Nakamura, Hidehiro Hojo, Masafumi Ikeda

**Affiliations:** 1 Department of Hepatobiliary and Pancreatic Oncology, National Cancer Center Hospital East, 6-5-1, Kashiwanoha, Kashiwa, Chiba Japan; 2 Department of Radiation Oncology and Particle Therapy, National Cancer Center Hospital East, 6-5-1, Kashiwanoha, Kashiwa, Chiba Japan

**Keywords:** Palliative radiotherapy, Bleeding, Pancreatic cancer, Hemostasis

## Abstract

**Background:**

Patients with unresectable pancreatic cancer (PC) sometimes experience gastrointestinal bleeding (GIB) due to tumor invasion of the gastrointestinal tract (tumor bleeding); no standard treatment has been established yet for this complication. Palliative radiotherapy (PRT) could be promising, however, there are few reports of PRT for tumor bleeding in patients with unresectable PC. Therefore, we evaluated the outcomes of PRT for tumor bleeding in patients with unresectable PC.

**Methods:**

We reviewed the medical records of patients with unresectable PC diagnosed at our institution between May 2013 and January 2022, and identified patients with endoscopically confirmed tumor bleeding who had received PRT. PRT was administered at a total dose of 30 Grays (Gy) in 10 fractions, 20 Gy in 5 fractions, or 8 Gy in a single fraction, and the dose selection was left to the discretion of the attending radiation oncologists.

**Results:**

During the study period, 2562 patients were diagnosed as having unresectable PC at our hospital, of which 225 (8.8%) developed GIB. Among the 225 patients, 63 (2.5%) were diagnosed as having tumor bleeding and 20 (0.8%) received PRT. Hemostasis was achieved in 14 of the 20 patients (70%) who received PRT, and none of these patients developed grade 3 or more adverse events related to the PRT. The median time to hemostasis was 8.5 days (range 7–14 days). The rebleeding rate was 21.4% (3/14). The median hemoglobin level increased significantly (*p* < 0.001) from 5.9 to 9.1 g/dL, and the median volume of red blood cell transfusion tended (*p* = 0.052) to decrease, from 1120 mL (range 280–3360 mL) to 280 mL (range 0–5560 mL) following the PRT. The median overall survival (OS) was 52 days (95% confidence interval [CI] 39–317). Of the 14 patients in whom hemostasis was achieved following PRT, chemotherapy could be started/resumed in seven patients (50%), and the median OS in these patients was 260 days (95% CI 76–not evaluable [NE]). Three patients experienced rebleeding (21.4%), on days 16, 22, and 25, after the start of PRT.

**Conclusion:**

This study showed that PRT is an effective and safe treatment modality for tumor bleeding in patients with unresectable PC.

**Supplementary Information:**

The online version contains supplementary material available at 10.1186/s13014-023-02367-5.

## Background

Pancreatic cancer (PC) is the seventh leading cause of cancer-related death worldwide [1], and the fourth leading cause of cancer death in Japan [2]. Although surgical resection is the only potentially curative treatment for PC, a substantial number of patients have unresectable PC at diagnosis [3]. A significant subset of patients with unresectable PC experience tumor-related complications such as weight loss, anorexia, abdominal pain, jaundice, and gastrointestinal bleeding (GIB). Among these, GIB is a relatively rare oncologic emergency, encountered in a reported approximately 1.6%-13% of patients, that can occasionally be life-threatening [4–6]. There are various causes of GIB in patients with PC, including peptic ulcer, variceal rupture, radiation-induced gastritis, and bleeding due to tumor invasion of the gastrointestinal tract (tumor bleeding). While endoscopic hemostasis has been established as the most suitable treatment for peptic ulcer, and arterial embolization for aneurysmal rupture, no standard treatment has been established yet for tumor bleeding. Inconsistent reports have been published on the effectiveness of endoscopic hemostasis for tumor bleeding, with the reported initial hemostasis rates ranging from 31%-40% and re-bleeding rates ranging from 16%-80% [7–9]. Therefore, endoscopic management of tumor bleeding still poses a challenge. Moreover, arterial embolization for tumor bleeding is also challenging, since this treatment modality is known to be effective for arterial bleeding, but not venous bleeding, which is the most common cause of tumor bleeding [10, 11]. Palliative radiotherapy (PRT) has sometimes been undertaken as a less-invasive option to treat tumor bleeding in patients with various other malignancies, and favorable outcomes have been reported [12, 13]. However, there are few reports of PRT undertaken for tumor bleeding in patients with unresectable PC. Therefore, we conducted this study to evaluate the outcomes of PRT for tumor bleeding in patients with unresectable PC.

## Methods

### Patients

We reviewed the medical records of patients diagnosed as having unresectable PC between May 2013 and January 2022 at our hospital, and all patients with locally advanced or metastatic PC with GIB who had received PRT were included in this study. Eastern Cooperative Oncology Group performance status (ECOG-PS) and palliative prognostic index (PPI) were used to evaluate the patient condition. PPI is a tool to predict the survival in patients with advanced cancers, and is calculated based on the palliative performance scale, amount of oral intake, and presence of edema, dyspnea at rest, and delirium. The PPI score ranges from 0 to 15, with higher scores indicating a worse survival prognosis, as follows: score of more than 6, less than three weeks life-expectancy; score of 4–6, three to six weeks life expectancy; score of less than 4, more than six weeks life expectancy [14]. This study was conducted with the approval of the Institutional Review Board of National Cancer Center Hospital East, Japan (No.2020–209), and in compliance with the principles laid down in the Declaration of Helsinki. Because this study was a retrospective observational study carried out in Japan, the need to obtain informed consent from the patients was waived by the Institutional Review Board of National Cancer Center Hospital East (No. 2020-209).

### Radiotherapy

**Fig. 1:**
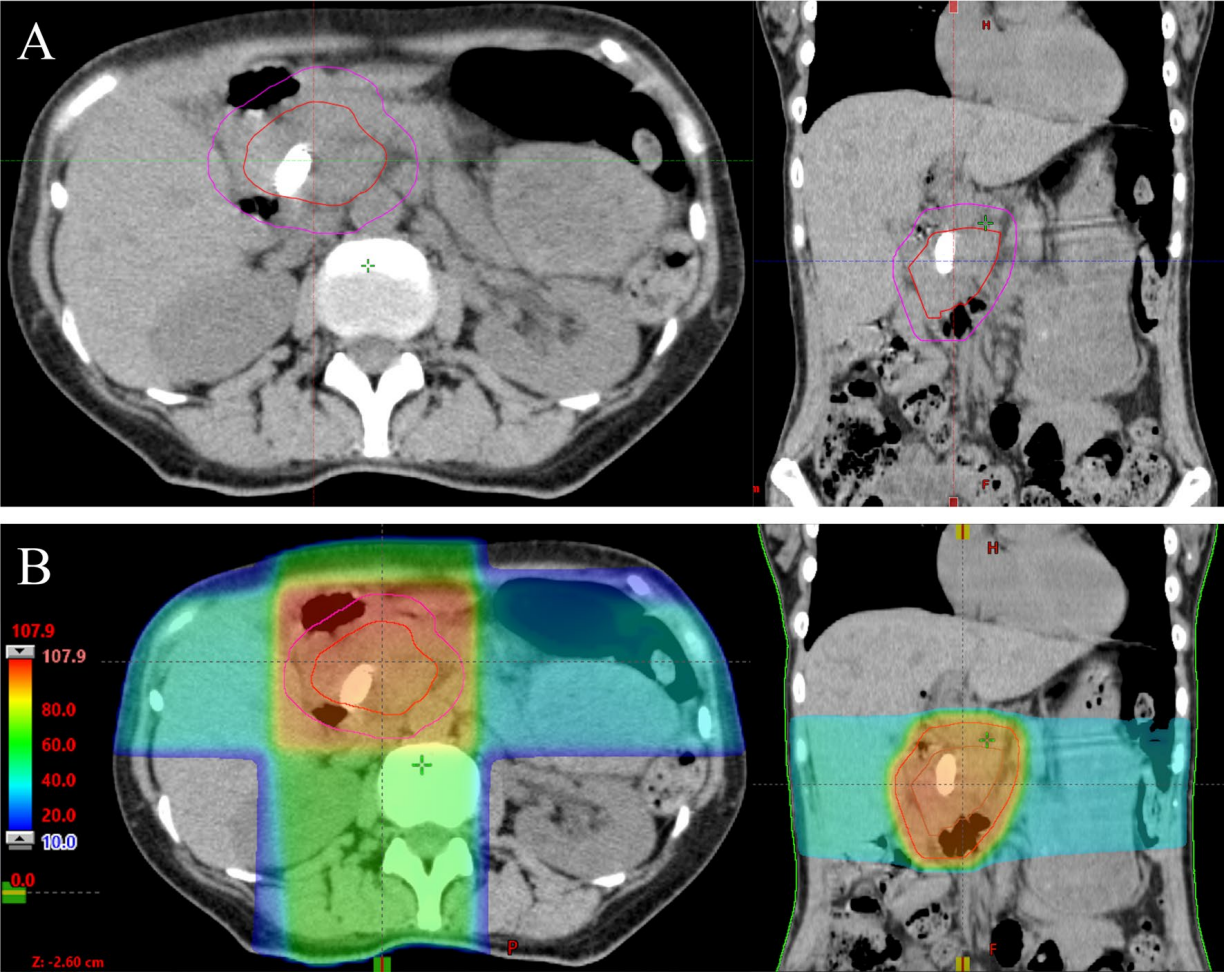
**A** Typical clinical target volume (red line) and planning target volume (purple line). **B** Dose distribution with clinical target volume (red line) and planning target volume (purple line)

PRT was performed at the discretion of the attending physicians and radiation oncologists. The planning for PRT was carried out using a 3-dimensional radiation planning system based on computed tomographic (CT) images. On the basis of the CT images, the clinical target volume (CTV) was determined as the whole tumor volume plus a sufficient margin around the tumor at the site of bleeding. The planning target volume (PTV) was defined as the CTV by expanding the CTV with a 1-cm margin (Fig. 1). After generating the PTV, the radiation oncologists devised a treatment plan that delivered an appropriate dose for PRT. The total radiation dose was 30 Grays (Gy) administered in 10 fractions, 20 Gy administered in 5 fractions, or 8 Gy administered in a single fraction, and the dose selection was left to the discretion of the treating radiation oncologist, based on the patient’s condition: in general, lower radiation doses were considered for patients who were in poor general condition.

### Definition and evaluation

Tumor bleeding was defined as the cause of the GIB when all of the following criteria were met: endoscopically confirmed tumor bleeding; hemoglobin (Hb) level < 7.0 g/dL or need for red blood cell (RBC) transfusion within four weeks prior to the PRT; patients with no bleeding from lesions other than PC. The day of hemostasis achievement was defined, based on previous reports [13], as the first day during the PRT when all of the following criteria (i)–(iii) had been satisfied for at least seven consecutive days: (i) increase of Hb level to ≥ 7.0 g/dL; (ii) no evidence of melena or hematemesis; and (iii) no necessity for blood transfusion. Time to hemostasis was defined as the period from the start of the PRT to the day of hemostasis achievement. We classified patients who needed additional hemostatic procedures as cases of failure of hemostasis. Rebleeding was defined as the need for RBC transfusion or additional hemostatic procedures after hemostasis was achieved with PRT. The Hb level was compared between its lowest value in the four weeks prior to the initiation of PRT and the one to four weeks after the initiation of PRT. The total volume of RBC transfusion was compared between the four weeks prior to the initiation of PRT and four weeks after the start of PRT. Adverse events were evaluated within four weeks of completion of the PRT according to the National Cancer Institute Common Terminology Criteria for Adverse Events version 4.0. Overall survival (OS) was defined as the period from the day of initiation of PRT to the day of death or final follow-up. OS was estimated using the Kaplan–Meier method, and the differences between groups were compared by the log-rank test. Hazard ratios (HR) were calculated using a Cox proportional hazards model. Continuous variables were compared using the Wilcoxon signed rank test or pairwise t-test Bonferroni correction, as appropriate. The Cochran-Armitage trend test was used to evaluate the associations between two variables. Differences at *P* < 0.05 were considered as being significant. The statistical analyses were performed using the software program R ver. 4.2.0 (R Foundation for Statistical Computing, Vienna, Austria).

## Results

### Patients

**Fig. 2:**
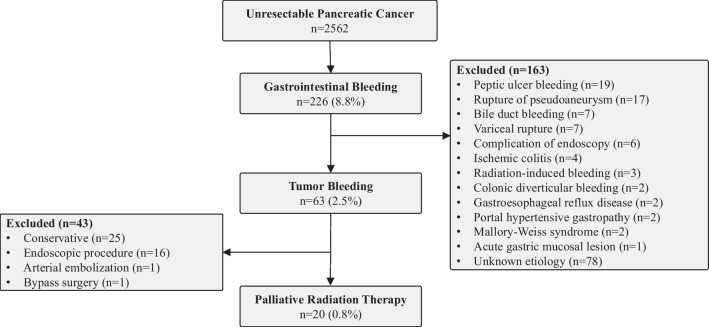
Flow diagram of enrollment of the study patients

During the study period, 2562 patients were diagnosed as having unresectable PC at our hospital, of which 225 patients (8.8%) developed GIB. Among these, 63 patients (2.5%) were diagnosed as having tumor bleeding and 20 (0.8%) received PRT for the control of tumor bleeding (Fig. 2). Table 1 shows the characteristics of the patients and the treatments administered. The median age at the time of tumor bleeding was 62 years (range 44–79 years), and 12 patients were male. The PPI score was 4 or higher in 14 patients (70%), and 17 patients (85%) had metastatic disease. All but one patient had symptoms, with tarry stool as the predominant symptom in the majority (50%). To confirm the bleeding site, every patient underwent gastrointestinal endoscopy, and contrast-enhanced CT was also performed prior to the PRT to rule out other causes of bleeding. Two patients were treated by duodenal stent placement and one patient received endoscopic hypertonic saline-epinephrine solution injection before the PRT, but none of these treatments led to successful hemostasis. Eight patients underwent biliary drainage via placement of a self-expandable metallic stent (SEMS) and one underwent biliary drainage via placement of a plastic stent (PS). Two patients underwent both biliary and duodenal placement of a SEMS. The characteristics of the patients who received conservative treatment without PRT (summarized in Additional file 1: Table S1) were compared with those who received PRT. These patients tended to be older, had poorer PS and PPI scores, and were more likely to have difficulty with further chemotherapy.

**Table 1:** Patient and treatment characteristics (n = 20)

Age	Years, median (range)	62 (44–79)
Sex, n (%)	Male	12 (60)
ECOG PS, n (%)	0	1 (5)
	1	4 (20)
	2	8 (40)
	3	5 (25)
	4	2 (10)
Palliative prognostic index score, n (%)	< 4 points	6 (30)
	4–6 points	8 (40)
	> 6 points	6 (30)
Location, n (%)	Head	12 (60)
	Body or tail	8 (40)
Extent of disease, n (%)	Locally advanced	3 (15)
	Metastatic	17 (85)
Histopathology, n (%)	Adenocarcinoma	18 (90)
	Anaplastic carcinoma	1 (5)
	Adenosquamous carcinoma	1 (5)
Initial symptom, n (%)	Tarry stool	10 (50)
	Hematochezia	3 (15)
	Hematemesis	2 (10)
	Abdominal pain	1 (5)
	Dizziness	1 (5)
	Fatigue	1 (5)
	Syncope	1 (5)
	None (incidentally endoscopically)	1 (5)
Bleeding site, n (%)	Duodenum	13 (65)
	Stomach	6 (30)
	Main pancreatic duct	1 (5)
Previous treatment, n (%)	Systemic chemotherapy	10 (50)
	None	10 (50)
Previous bleeding management, n (%)	None	17 (85)
	Duodenal stent	2 (10)
	Endoscopic injection of HSE	1 (5)
Previous biliary or duodenal stenting, n (%)	Biliary SEMS	8 (40)
	Biliary and duodenal SEMS	2 (10)
	Biliary plastic stent	1 (5)
	None	9 (45)
Radiation schedule, n (%)	8 Gy in a single fraction	2 (10)
	20 Gy in 5 fractions	14 (70)
	30 Gy in 10 fractions	4 (20)
Completion of PRT, n (%)	Yes	20 (100)

*ECOG PS* Eastern Cooperative Oncology Group performance status, *HSE* hypertonic saline epinephrine solution, *SEMS* self-expandable metallic stent, *Gy* gray, *PRT* palliative radiotherapy

### Treatment outcomes

The median follow-up period from the start of PRT was 41 days (range 15–372 days). The majority of patients (70%) received PRT at a total radiation dose of 20 Gy administered in 5 fractions, and the scheduled PRT regimen could be completed in all patients. Hemostasis was achieved in 14 patients (70%). The median time to hemostasis was 8.5 days (range 7–14 days). Classified by the radiation dose used for the PRT, the hemostasis rates were 100% (4/4), 64.3% (9/14), and 50% (1/2) in the patient groups administered PRT at 30 Gy, 20 Gy, and 8 Gy, respectively. Despite a tendency towards higher hemostasis rates with higher radiation doses, the differences were not statistically significant (*P* = 0.147). Of the six patients who failed to show hemostasis, four succumbed to tumor bleeding. In one of the remaining two patients, contrast-enhanced CT subsequently revealed bleeding from the first jejunal artery infiltrated by the tumor, and hemostasis was achieved by arterial embolization; the other of the two patients was transferred to receive palliative care. Of the 14 patients in whom hemostasis was achieved, three developed rebleeding (21.4%) 16, 22, and 25 days after the start of PRT. Out of these three patients, one died due to tumor bleeding 40 days after the initiation of PRT (18 days after rebleeding), in the second, hemostasis was achieved with gastrojejunal bypass, and the third patient was transferred to receive palliative care after failure of hemostasis.

**Fig. 3:**
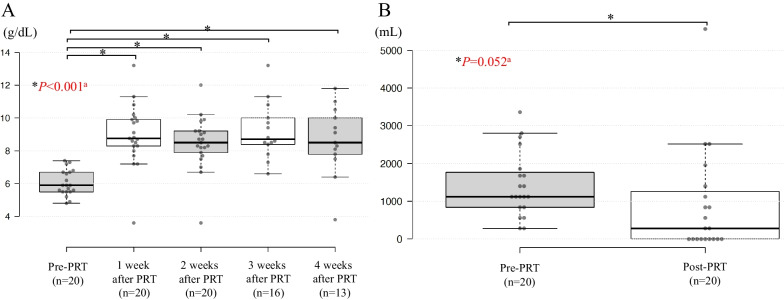
**A** Hemoglobin levels before and after palliative radiotherapy. **B** The volume of red blood cell transfusion before and after palliative radiotherapy

Prior to the PRT, the median Hb level was 5.9 g/dl (range 4.8–7.4). The median Hb level at 4 weeks after the start of PRT was 8.5 g/dl (range 3.8–11.8), being significantly higher as compared with the level recorded prior to the PRT (*P* < 0.001) (Fig. 3A). All patients had received RBC transfusions within four weeks prior to the PRT and the median volume of RBC transfusion decreased from 1120 mL (range 280–3360 mL) before the PRT to 280 mL (range 0–5560 mL) after the PRT, although the difference did not reach statistical significance (*P* = 0.052) (Fig. 3B).

**Fig. 4:**
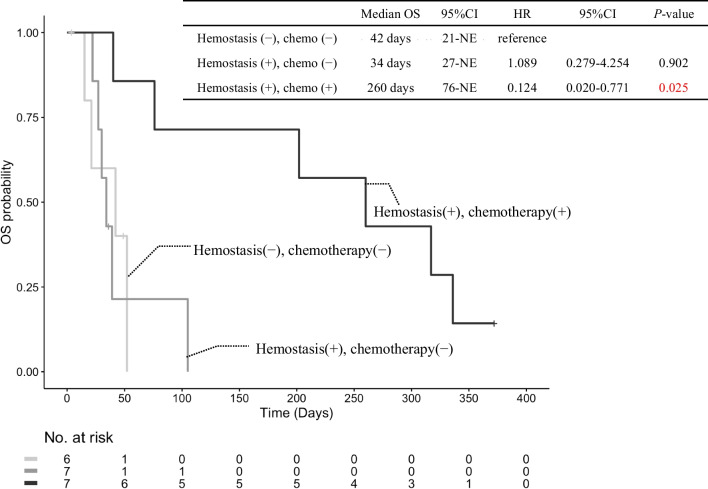
Kaplan–Meier curves of OS in patients with unresectable PC who received PRT for tumor bleeding. *OS* overall survival, *CI* confidence interval, *HR* hazard ratio, *NE* not evaluable

Out of the 20 patients who were treated by PRT, 15 died, with five of these patients succumbing to tumor bleeding and the remaining 10 to cancer progression. The median OS of the patients was 52 days (95% confidence interval [CI] 39–317). Of the 14 patients in whom hemostasis was achieved, chemotherapy could be initiated/resumed in seven patients, which led to a significantly improved survival as compared with that in patients in whom hemostasis was not achieved; the median OS in these patients was 260 days (HR 0.124; 95% CI 0.020–0.771). However, there was no statistically significant difference in the OS between the hemostasis-alone group and the group of patients in whom hemostasis was not achieved (Fig. 4).

### Adverse events

None of the patients included in this study who received PRT received concurrent chemotherapy. Systemic chemotherapy was initiated/ resumed in some patients after confirming hemostasis. All of the adverse events that were judged as having been induced by the PRT, including nausea, fatigue, anorexia, vomiting, and diarrhea, were classified as grade 1 or 2, and were manageable. There were no cases of grade 3 or more adverse events related to the PRT in this study. Grade 3 or 4 neutropenia, which was observed in patients, was judged as having been related to the concurrent chemotherapy. Grade 2 thrombocytopenia, which occurred in two patients, was attributed to disease progression in one patient and to infection in the other. Grade 4 thrombocytopenia occurred in one patient and was judged as having been caused by infection (Table 2).

**Table 2:** Adverse events (n = 20)

	Grade 1	Grade 2	Grade 3	Grade 4
Nausea, n (%)	10 (50)	1 (5)	0	0
Fatigue, n (%)	4 (20)	1 (5)	0	0
Anorexia, n (%)	2 (10)	0	0	0
Vomiting, n (%)	2 (10)	0	0	0
Diarrhea, n (%)	1 (5)	0	0	0
Neutropenia, n (%)	0	0	2 (10)^a^	1 (5)^a^
Thrombocytopenia, n (%)	0	2 (10)^b^	0	1 (5)^C^

^a^Grade 3 or 4 neutropenia was judged as being related to the concurrent chemotherapy

^b^Grade 2 thrombocytopenia was attributed to disease progression

^c^Grade 4 thrombocytopenia was judged as having been caused by infection

## Discussion

To the best of our knowledge, this is the first study to report the outcomes of PRT for tumor bleeding in patients with unresectable PC. More specifically, our findings demonstrated that PRT for tumor bleeding was an effective and safe treatment method, with a hemostasis achievement rate of 70% with no reports of grade 3 or more adverse events. In addition, we have included details about the cause of death of the patients and the post-PRT course. While we demonstrated that tumor bleeding could be fatal, we also showed that the high rate of achievement of hemostasis contributed to the initiation/resumption of chemotherapy after the PRT, which improved the survival prognosis of the patients.

In this study, 14 patients (70%) had a PPI score of 4 or higher, with a predicted survival of less than 6 weeks. However, the scheduled PRT could be completed in all the patients, with none showing grade 3 or more adverse events. This has the important significance that PRT is a feasible treatment option even for patients with a limited life expectancy.

Biliary or duodenal obstruction due to tumor invasion is a common complication of PC, and SEMS are often placed for treatment. Severe SEMS-related complications, such as GIB, have been reported to be associated with chemoradiotherapy following SEMS placement for PC [15]. Thus, caution should be exercised when considering radiotherapy after SEMS placement for patients with advanced PC. In our study, SEMS had been placed for biliary drainage in ten patients, including two who underwent both biliary and duodenal stenting with SEMS before PRT. However, no stent-related adverse events were observed in any of these patients.

The most reliable method to confirm hemostasis after PRT is direct endoscopic observation, but as this could be challenging in patients in poor general condition, varying definitions of hemostasis have been adopted in previous studies. In this study, endoscopy after completion of PRT could be performed in only seven patients (35%). Therefore, we defined hemostasis based on the objective measurements described above, as in a previous study [13]. In our present study, hemostasis was achieved in 14 out of 20 patients (70%), which was consistent with previous reports from studies conducted in gastric cancer patients of rates ranging from 54 to 73% [13, 16–18]. Aguilera ML et al., who performed a comprehensive study of the causes and treatments of GIB in PC patients, reported a hemostasis rate following PRT of 100% (8/8) [4]. However, in their study, hemostasis was defined solely based on the reduced volume of RBC transfusion needed after PRT as compared with that before PRT; since the transfusion volume could be expected to vary depending on various factors, such as the primary treatment strategy, careful interpretation of this finding is necessary. We also demonstrated that the median time to hemostasis was 8.5 days (range 7–14). Although hemostasis was achieved within 10 days in the majority of patients, in two patients, it took 14 days before PRT exerted its hemostatic effect. Thus, it is worth noting that in some patients, achievement of hemostasis in response to PRT could be delayed.

The relationship between the total radiation dose and the likelihood of successful hemostasis has been evaluated in several studies. Tey et al. found no significant difference in the hemostasis achievement rate among patient groups who received PRT at different total radiation doses [19]. On the other hand, some studies have indicated that use of higher radiation doses for PRT may be more effective [16, 20, 21]. In our study, the hemostasis rates were 100%, 64%, and 50% in patients who received PRT at 30 Gy, 20 Gy, and 8 Gy, respectively. Although there was a trend towards higher hemostasis rates at higher radiation doses, the differences were not statistically significant. Thus, the relationship between the radiation dose and hemostasis efficacy must be interpreted with caution.

We also demonstrated that PRT improved the anemia and reduced the volume of RBC transfusion. In addition, chemotherapy could be initiated/resumed in half of the patients in whom hemostasis was achieved with PRT, which led to a significantly improved survival. However, there was no statistically significant difference in the OS between the hemostasis-alone group and the group of patients in whom hemostasis was not achieved, possibly because most of the patients had a limited life expectancy even at the time of initiation of the PRT. According to previous studies conducted in patients with gastric cancer, the rebleeding rate in patients in whom hemostasis is initially achieved with PRT is in the range of 25–50% [16–18, 20, 22]. In our cohort, rebleeding occurred in three out of the 14 patients (21.4%) in whom hemostasis was initially achieved, which was consistent with previous reports. All the three patients had received PRT at 20 Gy administered in 5 fractions.

Some limitations of this study need to be mentioned here. First, this study was a retrospective study conducted at a single center, which could have introduced biases. However, to make this study more meaningful and informative, we adopted strict definitions for tumor bleeding and hemostasis, and investigated the causes of death of the patients. Second, we could not include data such as quality of life (QOL) before and after treatment, because of the retrospective nature of the study. Therefore, the impact of PRT on the QOL of the patients remains to be clarified. Finally, the radiation dose for the PRT was determined at the discretion of the radiation oncologists, depending on the patient condition. This could have led to a lower radiation dose being used for patients in poorer general condition, and consequently, a biased interpretation of the hemostatic effect of PRT. To elucidate this issue more clearly and to eliminate the effects of confounding factors, analysis of data from a larger cohort is necessary. Despite the aforementioned limitations, we believe that our findings have important implications in the management of this rare condition, as there are no previous reports on the outcomes of PRT used for tumor bleeding in patients with unresectable PC.

## Conclusion

PRT was an effective and safe treatment modality for tumor bleeding in patients with unresectable PC.

### Supplementary Information

**Additional file 1. Supplementary table 1.** Characteristics of the patients who were treated with PRT and conservative treatment only.

## Data Availability

Data from this study will be made available by the corresponding author upon reasonable request.

## Abbreviations

PC: Pancreatic cancer
GIB: Gastrointestinal bleeding
PRT: Palliative radiotherapy
Hb: Hemoglobin
RBC: Red blood cell
Gy: Gray
ECOG–PS: Eastern Cooperative Oncology Group performance status
PPI: Palliative prognostic index
CT: Computed tomography
OS: Overall survival
HR: Hazard ratio
CI: Confidence interval
NE: Not evaluable
QOL: Quality of life

## References

[1] Bray F, Ferlay J, Soerjomataram I, Siegel RL, Torre LA, Jemal A (2018). Global cancer statistics 2018: GLOBOCAN estimates of incidence and mortality worldwide for 36 cancers in 185 countries. CA Cancer J Clin.

[2] Hori MMT, Shibata A, Katanoda K, Sobue T, Nishimoto H (2015). Cancer incidence and incidence rates in Japan in 2009: a study of 32 population-based cancer registries for the Monitoring of Cancer Incidence in Japan (MCIJ) project. Jpn J Clin Oncol.

[3] Butturini G, Stocken DD, Wente MN, Jeekel H, Klinkenbijl JH, Bakkevold KE, Takada T, Amano H, Dervenis C, Bassi C (2008). Influence of resection margins and treatment on survival in patients with pancreatic cancer: meta-analysis of randomized controlled trials. Arch Surg.

[4] Aguilera Munoz L, de Mestier L, Lamallem H, Jaïs B, Maire F, Lévy P, Rebours V, Hammel P (2020). Gastrointestinal bleeding in patients with pancreatic cancer: causes and haemostatic treatments. United European Gastroenterol J.

[5] Wang YU, Yuan C, Liu X (2015). Characteristics of gastrointestinal hemorrhage associated with pancreatic cancer: a retrospective review of 246 cases. Mol Clin Oncol.

[6] Tempero MA, Malafa MP, Al-Hawary M, Asbun H, Bain A, Behrman SW, Benson AB, Binder E, Cardin DB, Cha C (2017). Pancreatic adenocarcinoma, version 2.2017, NCCN clinical practice guidelines in oncology. J Natl Compr Canc Netw.

[7] Suzuki H, Miho O, Watanabe Y, Kohyama M, Nagao F (1989). Endoscopic laser therapy in the curative and palliative treatment of upper gastrointestinal cancer. World J Surg.

[8] Mathus-Vliegen EM, Tytgat GN (1990). Analysis of failures and complications of neodymium: YAG laser photocoagulation in gastrointestinal tract tumors. A retrospective survey of 18 years' experience. Endoscopy.

[9] Adler DG, Leighton JA, Davila RE, Hirota WK, Jacobson BC, Qureshi WA, Rajan E, Zuckerman MJ, Fanelli RD, Hambrick RD (2004). ASGE guideline: the role of endoscopy in acute non-variceal upper-GI hemorrhage. Gastrointest Endosc.

[10] Gwon DI, Ko GY, Sung KB, Shin JH, Kim JH, Yoon HK (2011). Endovascular management of extrahepatic artery hemorrhage after pancreatobiliary surgery: clinical features and outcomes of transcatheter arterial embolization and stent-graft placement. AJR Am J Roentgenol.

[11] Limongelli P, Khorsandi SE, Pai M, Jackson JE, Tait P, Tierris J, Habib NA, Williamson RCN, Jiao LR (2008). Management of delayed postoperative hemorrhage after pancreaticoduodenectomy: a meta-analysis. Arch Surg.

[12] Sapienza LG, Ning MS, Jhingran A, Lin LL, Leao CR, da Silva BB, Pellizzon ACA, Gomes MJL, Baiocchi G (2019). Short-course palliative radiation therapy leads to excellent bleeding control: a single centre retrospective study. Clin Transl Radiat Oncol.

[13] Kondoh C, Shitara K, Nomura M, Takahari D, Ura T, Tachibana H, Tomita N, Kodaira T, Muro K (2015). Efficacy of palliative radiotherapy for gastric bleeding in patients with unresectable advanced gastric cancer: a retrospective cohort study. BMC Palliat Care.

[14] Morita T, Tsunoda J, Inoue S, Chihara S (1999). The Palliative Prognostic Index: a scoring system for survival prediction of terminally ill cancer patients. Support Care Cancer.

[15] Das R, Hadley S, Sahaibbs V, Bednar F, Evans J, Lawrence T, Cuneo K (2023). Predictors of acute and late toxicity in patients receiving chemoradiation for unresectable pancreatic cancer. Adv Radiat Oncol.

[16] Tey J, Back MF, Shakespeare TP, Mukherjee RK, Lu JJ, Lee KM, Wong LC, Leong CN, Zhu M (2007). The role of palliative radiation therapy in symptomatic locally advanced gastric cancer. Int J Radiat Oncol Biol Phys.

[17] Hashimoto K, Mayahara H, Takashima A, Nakajima TE, Kato K, Hamaguchi T, Ito Y, Yamada Y, Kagami Y, Itami J (2009). Palliative radiation therapy for hemorrhage of unresectable gastric cancer: a single institute experience. J Cancer Res Clin Oncol.

[18] Asakura H, Hashimoto T, Harada H, Mizumoto M, Furutani K, Hasuike N, Matsuoka M, Ono H, Boku N, Nishimura T (2011). Palliative radiotherapy for bleeding from advanced gastric cancer: is a schedule of 30 Gy in 10 fractions adequate?. J Cancer Res Clin Oncol.

[19] Tey J, Choo BA, Leong CN, Loy EY, Wong LC, Lim K, Lu JJ, Koh WY (2014). Clinical outcome of palliative radiotherapy for locally advanced symptomatic gastric cancer in the modern era. Medicine (Baltimore).

[20] Kim MM, Rana V, Janjan NA, Das P, Phan AT, Delclos ME, Mansfield PF, Ajani JA, Crane CH, Krishnan S (2008). Clinical benefit of palliative radiation therapy in advanced gastric cancer. Acta Oncol.

[21] Lee YH, Lee JW, Jang HS (2017). Palliative external beam radiotherapy for the treatment of tumor bleeding in inoperable advanced gastric cancer. BMC Cancer.

[22] Chaw CL, Niblock PG, Chaw CS, Adamson DJ (2014). The role of palliative radiotherapy for haemostasis in unresectable gastric cancer: a single-institution experience. Ecancermedicalscience.

