# Correction: Palliative radiotherapy for tumor bleeding in patients with unresectable pancreatic cancer: a single-center retrospective study

**DOI:** 10.1186/s13014-023-02377-3

**Published:** 2023-12-12

**Authors:** Taro Shibuki, Mitsuhito Sasaki, Shota Yamaguchi, Kanae Inoue, Tomonao Taira, Tomoyuki Satake, Kazuo Watanabe, Hiroshi Imaoka, Shuichi Mitsunaga, Takeshi Fujisawa, Kento Tomizawa, Hidekazu Oyoshi, Masaki Nakamura, Hidehiro Hojo, Masafumi Ikeda

**Affiliations:** 1 Department of Hepatobiliary and Pancreatic Oncology, National Cancer Center Hospital East, 6-5-1, Kashiwanoha, Kashiwa, Chiba Japan; 2 Department of Radiation Oncology and Particle Therapy, National Cancer Center Hospital East, 6-5-1, Kashiwanoha, Kashiwa, Chiba Japan

**Radiation Oncology (2023) 18:178**

10.1186/s13014-023-02367-5

After publication of this article [1], the authors reported that in this article the graphics relating to Figs. 1, 2, 3 and 4 captions had been interchanged; the figures should have appeared as shown below.

**Fig. 1:**
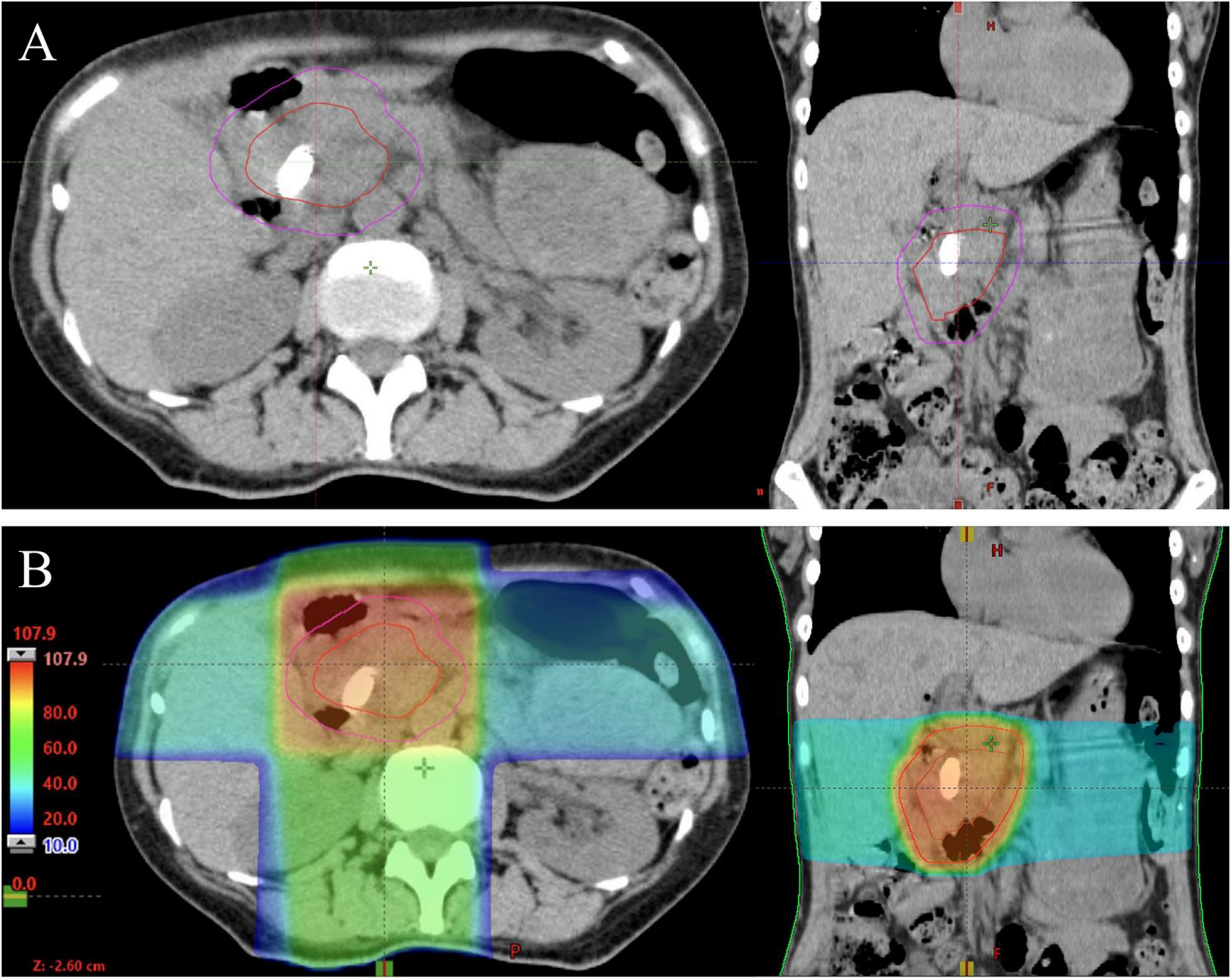
**A** Typical clinical target volume (red line) and planning target volume (purple line). **B** Dose distribution with clinical target volume (red line) and planning target volume (purple line)

**Fig. 2:**
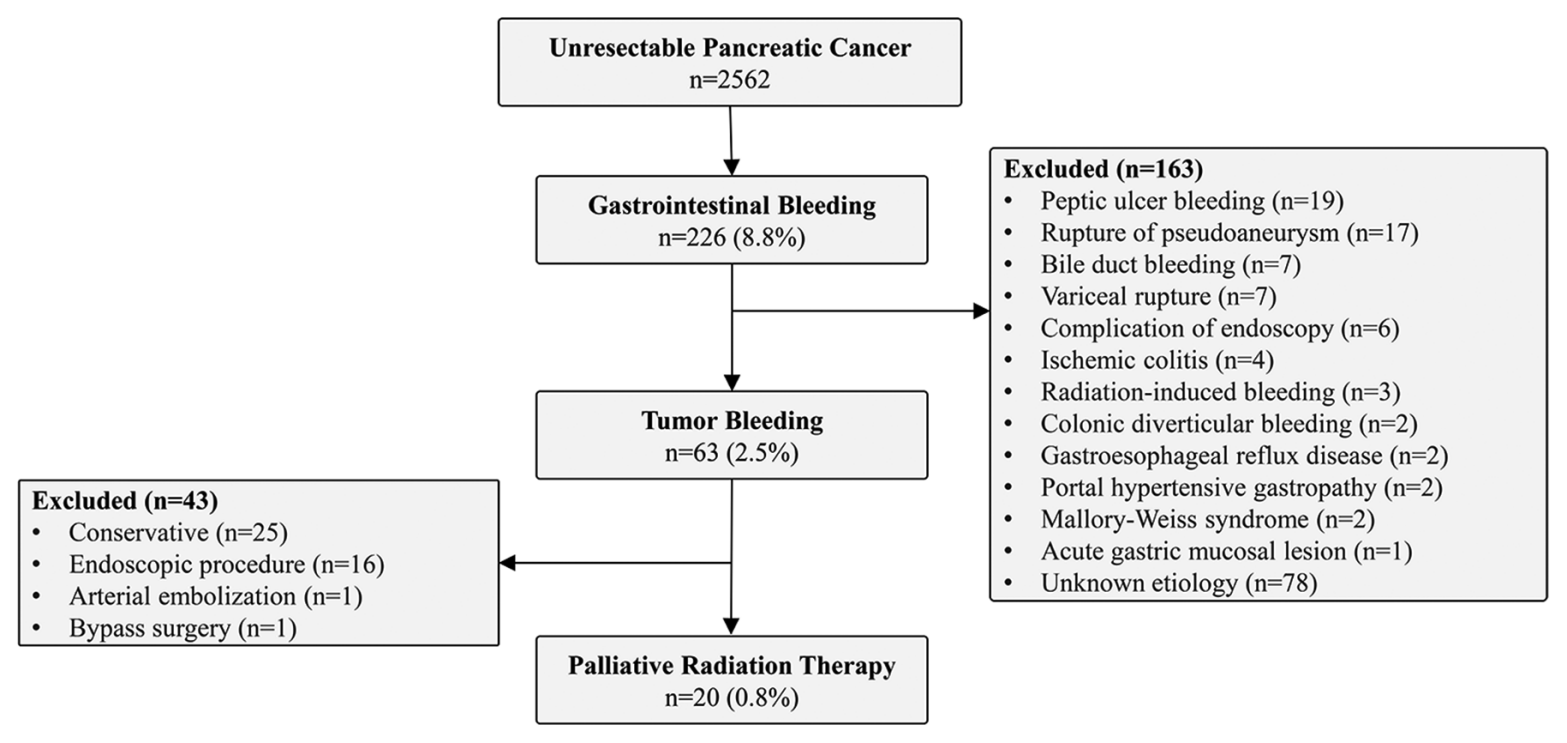
Flow diagram of enrollment of the study patients

**Fig. 3:**
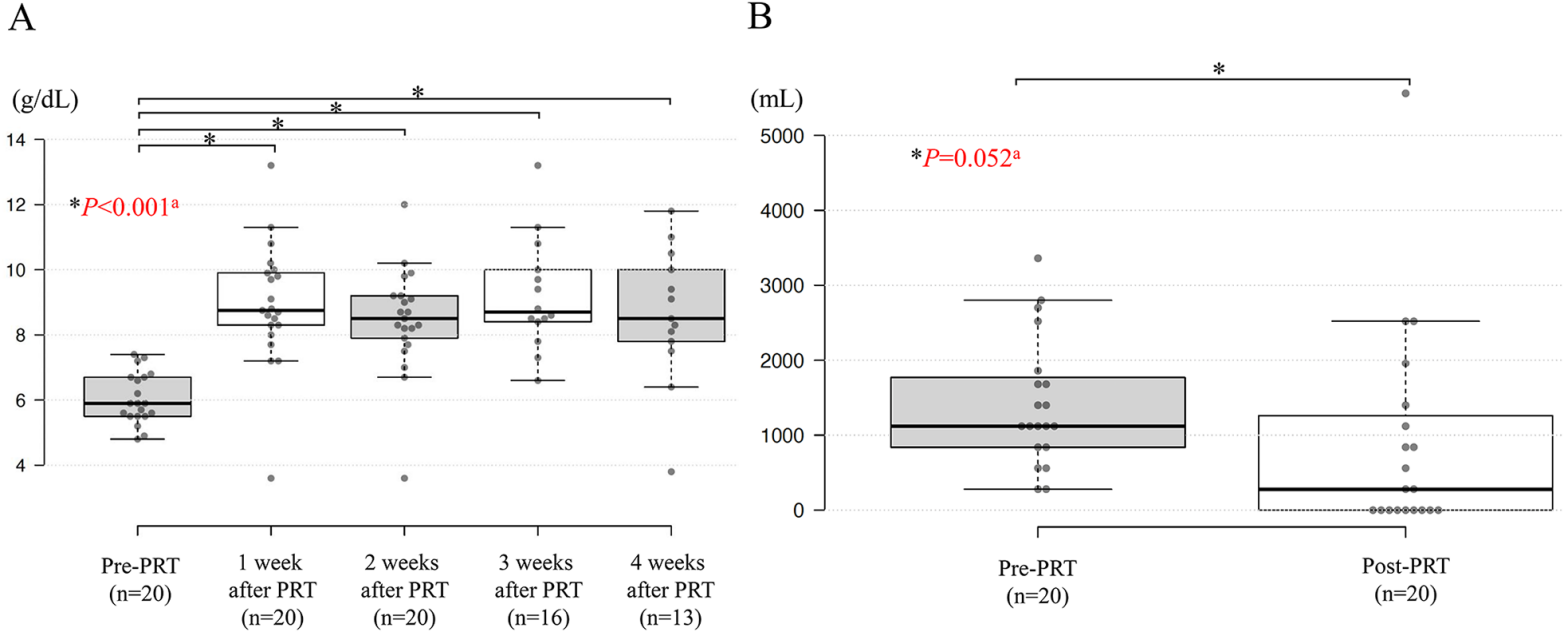
**A** Hemoglobin levels before and after palliative radiotherapy. **B** The volume of red blood cell transfusion before and after palliative radiotherapy

**Fig. 4:**
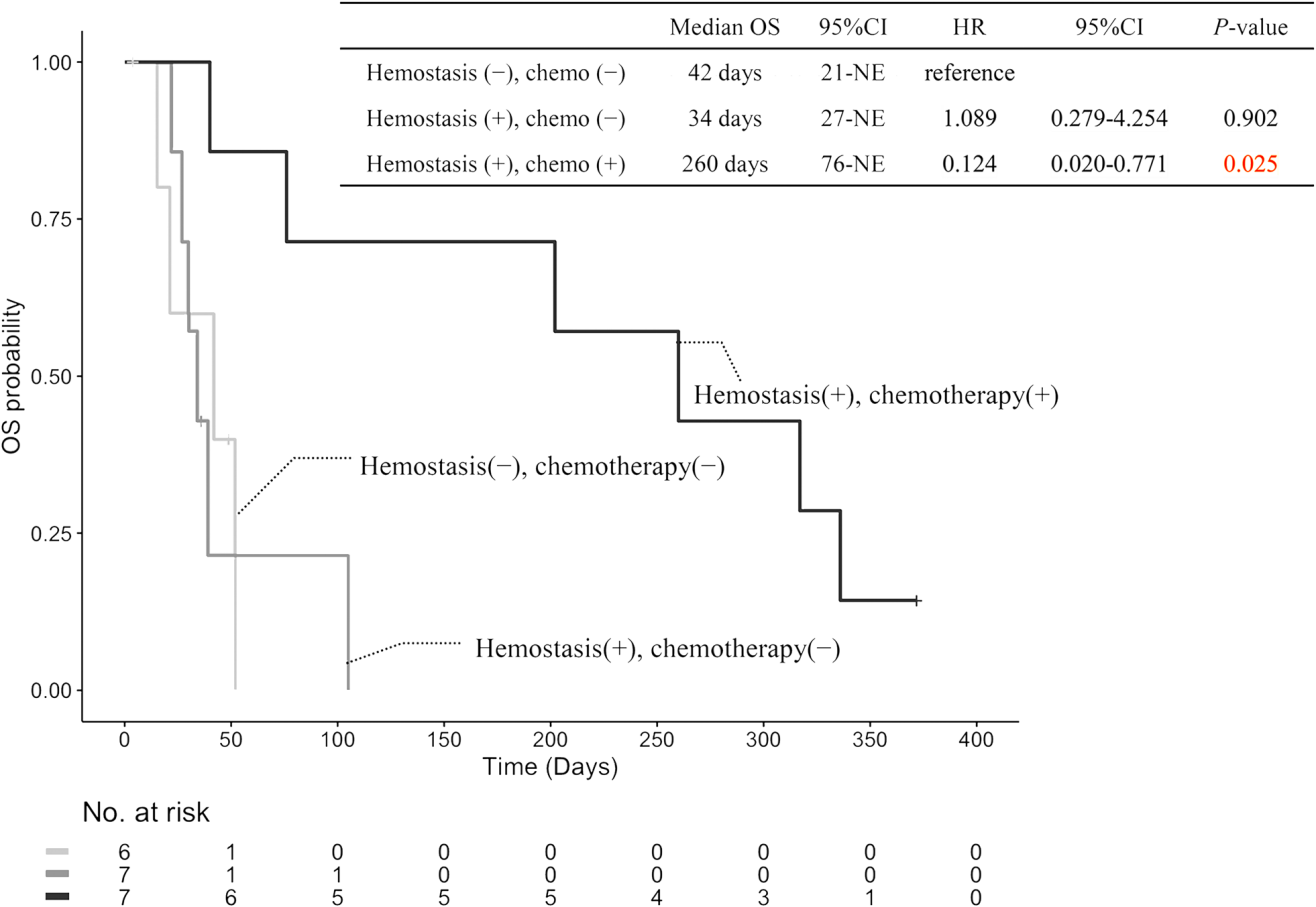
Kaplan?Meier curves of OS in patients with unresectable PC who received PRT for tumor bleeding. *OS* overall survival, *CI* confidence interval, *HR* hazard ratio, *NE* not evaluable

The original article [1] has been corrected.
