# Supplementary material for: Palliative radiotherapy for tumor bleeding in patients with unresectable pancreatic cancer: a single-center retrospective study
Source: Radiat Oncol. 2023 Oct 31;18:178. doi: 10.1186/s13014-023-02367-5 (PMC10617159; doi:10.1186/s13014-023-02367-5)
Supplement: Supplementary file 1 — Additional file 1. Supplementary table 1. Characteristics of the patients who were treated with PRT and conservative treatment only. [file 13014_2023_2367_MOESM1_ESM.docx]

| **Supplementary table 1. Characteristics of the patients who were treated with PRT and conservative treatment only.** | | | | |
| --- | --- | --- | --- | --- |
|  |  | PRT  (n=20) | Conservative treatment only  (n=25) | *P*-value |
| Age | Years, median (range) | 62 (44-79) | 70 (50-85) | 0.120 |
| Sex, n (%) | Male | 12 (60) | 18 (72) | <0.001 |
| ECOG PS, n (%) | 0 | 1 (5) | 1 (4) | 0.078 |
|  | 1 | 4 (20) | 4 (16) |  |
|  | 2 | 8 (40) | 2 (8) |  |
|  | 3 | 5 (25) | 10 (40) |  |
|  | 4 | 2 (10) | 8 (32) |  |
| Palliative prognostic index score, n (%) | < 4 points | 6 (30) | 5 (20) | 0.126 |
|  | 4-6 points | 8 (40) | 5 (20) |  |
|  | >6 points | 6 (30) | 15 (60) |  |
| Location, n (%) | Head | 12 (60) | 12 (48) | 0.616 |
|  | Body or tail | 8 (40) | 13 (52) |  |
| Extent of disease, n (%) | Locally advanced | 3 (15) | 3 (12) | 1 |
|  | Metastatic | 17 (85) | 22 (88) |  |
| Histopathology, n (%) | Adenocarcinoma | 18 (90) | 21 (84) | 0.550 |
|  | Adenosquamous carcinoma | 1 (5) | 2 (8) |  |
|  | Anaplastic carcinoma | 1 (5) | 0 |  |
|  | Carcinoma | 0 | 1 (4) |  |
|  | Unknown | 0 | 1 (4) |  |
| Initial symptom, n (%) | Tarry stool | 10 (50) | 3 (12) | 0.061 |
|  | Hematochezia | 3 (15) | 10 (40) |  |
|  | Hematemesis | 2 (10) | 7 (28) |  |
|  | Fatigue | 1 (5) | 2 (8) |  |
|  | None (incidentally endoscopically) | 1 (5) | 3 (12) |  |
|  | Abdominal pain | 1 (5) | 0 |  |
|  | Dizziness | 1 (5) | 0 |  |
|  | Syncope | 1 (5) | 0 |  |
| Bleeding site, n (%) | Duodenum | 13 (65) | 12 (48) | 0.91 |
|  | Stomach | 6 (30) | 10 (40) |  |
|  | Main pancreatic duct | 1 (5) | 0 |  |
|  | Colon | 0 | 3 (12) |  |
| Previous treatment, n (%) | Systemic chemotherapy | 10 (50) | 23 (92) | 0.005 |
|  | None | 10 (50) | 2 (8) |  |
| Previous biliary or duodenal stenting, n (%) | Biliary SEMS | 8 (40) | 6 (24) | 0.085 |
|  | Biliary and duodenal SEMS | 2 (10) | 1 (4) |  |
|  | Biliary plastic stent | 1 (5) | 0 |  |
|  | Duodenal SEMS | 0 | 4 (16) |  |
|  | None | 9 (45) | 14 (56) |  |
| Reason for conservative treatment only, n (%) | Poor general condition or serious comorbidities | - | 14 (56) | - |
|  | No progression of anemia observed after fasting and proton pomp inhibitor | - | 11 (44) |  |

PRT, palliative radiotherapy; ECOG PS, Eastern Cooperative Oncology Group performance status; SEMS, self-expandable metallic stent
